# Validation of the brief assessment of impaired cognition (BASIC) and brief assessment of impaired cognition questionnaire (BASIC-Q) in Italy

**DOI:** 10.1007/s10072-025-08691-w

**Published:** 2026-03-09

**Authors:** Marco Canevelli, Simone Pomati, Emanuela Salati, Giorgia Maestri, Giulia Sarti, Clara Calia, Chiara Pecorari, Sara Marchetti, Ilaria Cova, T. Rune Nielsen, Kasper Jørgensen, Daniel Kjaergaard, Francesco Sciancalepore, Filippo Nuti, Marco Toccaceli Blasi, Leonardo Pantoni, Nicola Vanacore, Giuseppe Bruno

**Affiliations:** 1 Department of Human Neuroscience, Sapienza University, Rome, Italy; 2 National Center for Disease Prevention and Health Promotion, Italian National Institute of Health, Rome, Italy; 3 Aging Research Center, Department of Neurobiology, Care Sciences and Society, Karolinska Institutet and Stockholm University, Stockholm, Sweden; 4 Istituti Clinici Scientifici Maugeri IRCCS, Pavia, Italy; 5 Neurology Unit, Luigi Sacco University Hospital, Milan, Italy; 6 School of Health in Social Science, University of Edinburgh, Edinburgh, UK; 7 Danish Dementia Research Centre, Department of Neurology, Copenhagen University Hospital -Rigshospitalet, Copenhagen, Denmark; 8 Department of Psychology, University of Copenhagen, Copenhagen, Denmark; 9 Barcelonaβeta Brain Research Center (BBRC), Pasqual Maragall Foundation, Barcelona, Spain; 10 Department of Clinical and Biomedical Sciences, Neuroscience Research Center, University of Milan, Milan, Italy; 11 Department of Neurorehabilitation Sciences, Casa di Cura Igea, Milan, Italy

**Keywords:** Cognitive impairment, Cognitive assessment, Dementia, Culturally and linguistically diverse, Cross-cultural neuropsychology

## Abstract

**Background:**

Brief and accurate tools are needed to enhance the prompt identification of cognitive disorders. This study aimed to translate and adapt two recently developed, highly accurate instruments into Italian: the Brief Assessment of Impaired Cognition (BASIC) and the BASIC Questionnaire (BASIC-Q), and to validate their diagnostic accuracy.

**Methods:**

The Italian versions of BASIC and BASIC-Q were developed following a rigorous process and were validated in patient populations referred to two Italian tertiary memory clinics. Receiver operating characteristic curve analysis was used to evaluate the discriminative validity of BASIC and BASIC-Q in identifying cognitive impairment compared to specialist diagnoses. Linear regression models were used to explore the influence of traditional confounders, such as age and education.

**Results:**

A total of 246 participants were recruited for the study. The BASIC and BASIC-Q assessments accurately distinguished between individuals with cognitive impairment and those with intact cognition, achieving areas under the curve of 0.96 and 0.95, respectively. The accuracy of these two tools was comparable to that of the Mini-Mental State Examination. Additionally, both instruments showed a negative association with age but were not influenced by educational level.

**Discussion:**

The BASIC and BASIC-Q tools effectively identify older individuals with cognitive impairment who may need further diagnostic assessment. Their accuracy must be explored in primary care settings and among multicultural populations.

**Supplementary Information:**

The online version contains supplementary material available at 10.1007/s10072-025-08691-w.

## Introduction

 The prompt and accurate diagnosis of cognitive disorders is of utmost importance, as it plays a critical role in ensuring individuals have access to dedicated healthcare resources and receive the necessary interventions. Early detection of cognitive impairment enables the identification and treatment of reversible factors and causes, allowing for timely interventions that can improve cognitive functioning. It supports the implementation of lifestyle interventions to promote brain/cognitive health and slow decline. Moreover, it also enables the creation of patient-centered treatment plans and facilitates access to emerging therapies and clinical trials [1]. These considerations underscore the critical importance of developing brief and precise tools for identifying cognitive disorders [2]. These instruments are needed in specialized clinical services. Still, they are particularly required in primary care and community settings, as these often serve as the first point of contact for individuals with cognitive impairments [1–3].

 A wide range of tests is currently available for screening cognitive disorders, with good psychometric properties [4]. However, some of the most commonly used brief cognitive assessments, such as the Mini-Mental State Examination (MMSE) [5] and Montreal Cognitive Assessment [6], are significantly influenced by sociodemographic factors like age and education, which complicates their application in clinical practice [7, 8]. Some instruments, including the MMSE, lack sensitivity for mild dementia and mild cognitive impairment (MCI) [4, 9]. Other test and brief test batteries, such as the Addenbrooke’s Cognitive Examination Revised [10], are relatively time-consuming and can hardly be routinely adopted in busy clinical settings. Only a few tools, such as the General Practitioner assessment of Cognition (GPCOG) [11], integrate cognitive tests with informants’ or patients’ reports. However, this combination has been shown to increase diagnostic accuracy [12]. Finally, most of the available tests are affected by cultural biases and may fail to provide a reliable assessment among culturally and linguistically diverse (CALD) individuals [13]. This latter consideration assumes special relevance in light of the increasing number of migrants and older people from ethnic minorities seeking help for cognitive disturbances in host countries [14].

 Based on these premises, two tools have recently been developed to identify cognitive impairment within primary care and community settings: the Brief Assessment of Impaired Cognition (BASIC) and the BASIC Questionnaire (BASIC-Q) [15, 16]. Both instruments can be administered in approximately five minutes, meeting the need for quick assessments in these environments. The BASIC and BASIC-Q were designed from a cross-cultural perspective, incorporating tasks that have high discriminative validity and minimal cultural, linguistic, and educational bias. BASIC and BASIC-Q have already shown excellent accuracy in detecting dementia and MCI in both memory clinics and primary care [15–20]. Recently, their diagnostic accuracy has been confirmed in a multicultural memory clinic population of 479 patients across six European countries [21]. They have also already been translated into other languages without the need for any cultural adaptation [22, 23].

The current study aimed to translate and adapt the BASIC and BASIC-Q questionnaires into Italian and assess their accuracy in a population of both cognitively intact and cognitively impaired individuals referred to two Italian Centers for Cognitive Disorders and Dementias (CCDDs).

## Methods

### Participants and setting

 In the current study, patients who were referred for cognitive disturbances to two tertiary Cognitive Disorders and Dementias Centers (CCDDs) in Italy were consecutively enrolled. The CCDDs were affiliated with (1) the Department of Human Neuroscience, Policlinico Umberto I University Hospital, Rome, and (2) the Luigi Sacco University Hospital, Milan. Participants accessed the two CCDDs through standard clinical pathways, specifically following referrals from their general practitioners or other specialists. Patients with a migration background (i.e., living in Italy but born abroad) were included in the study, provided they had sufficient proficiency in Italian (i.e., they commonly used the Italian language and demonstrated a clear understanding of the test instructions). Exclusion criteria included severe psychiatric symptoms (e.g., severe depression, psychosis) and a diagnosis other than dementia, MCI, or subjective cognitive decline (SCD). It is worth noting that data from 221 (89.8%) of the enrolled participants had already been included in a multicenter study aimed at validating the BASIC and BASIC-Q in a multicultural memory clinic sample across six European countries [21]. However, detailed information on the accuracy of these tools in the Italian subsample of participants had not been provided.

### BASIC and BASIC-Q

The BASIC consists of four components: (1) Self-Report, (2) Supermarket Fluency, (3) Category Cued Memory Test (CCMT), and (4) Informant-Report [15]. The total score ranges from 0 to 25 points, where lower scores indicate more severe cognitive impairment.

The BASIC-Q is a short questionnaire consisting of 10 items divided into three sections: (1) Self-Report, (2) Orientation, and (3) Informant-Report [16]. The total score ranges from 0 to 20 points, with lower scores indicating higher degrees of cognitive impairment.

If a reliable informant report is unavailable, prorated scores can be used for both instruments.

A broader description of BASIC and BASIC-Q is provided in Table 1.

**Table 1:** Description of the brief assessment of impaired cognition (BASIC) and brief assessment of impaired cognition questionnaire (BASIC-Q)

BASIC			BASIC-Q		
Component	Description	Score range	Component	Description	Score range
***1. Self-Report***	• Compared with previously, do you feel that your memory has declined substantially? • Do you need more help from others to remember appointments, family occasions, or holidays? • Do you have more trouble recalling names, finding the right words, or completing sentences?	0–6	***1. Self-Report***	Identical to BASIC	0–6
***2. Supermarket Fluency***	The patient is asked to name as many supermarket items as he or she can think of in 1 min	0–5	***2. Orientation***	Orientation in time (year, month, day of week) and orientation in person (age)	0–8
***3. Category Cued Memory Test***	Four pictures are connected to specific semantic categories (banana ↔ fruit; cow ↔ animal; sofa ↔ furniture; bicycle ↔ means of transportation) by forced choice. After 2 min of distraction, the patient is asked to freely recall the objects. If one or more objects are not retrieved by free recall, the examiner provides the relevant semantic cue (e.g., “There was also a fruit. Which fruit was it?”).	0–8	***3. Informant Report***	Identical to BASIC	0–6
***4. Informant Report***	Compared with a few years ago, how is your spouse/parent/relative/this person at: • Remembering things that have happened recently? • Recalling conversations a few days later? • Remembering what day and month it is?	0–6			
***Total score***		0–25	***Total score***		0–20

Adapted and modified from [21]

### Development of the Italian versions of the BASIC and BASIC-Q

The Italian versions of the two instruments were developed in compliance with the neuropsychological application of the TD-1 and TD-2 guidelines of the International Test Commission Guidelines for Translation and Adapting of Tests [24]. Specifically, after obtaining permission from the intellectual property rights owner (i.e., the Danish Dementia Research Center, Copenhagen, Denmark), the two tools were translated into Italian and back into the original English version by two pairs of independent bilingual and bicultural translators. The two back-translated versions were then compared with the original versions by a multidisciplinary committee, including one epidemiologist, two healthcare professionals experienced in cognitive assessment, all four translators involved in these two first stages, and the developers of the instruments. No structural or format changes of any of the BASIC and BASIC-Q items were needed during translation. The final Italian versions of the BASIC and BASIC-Q are provided as Online Resources.

### Validation of the Italian versions of the BASIC and BASIC-Q

All participants underwent a comprehensive medical history interview. The BASIC and BASIC-Q were administered alongside the MMSE [5] by trained neuropsychologists who were blinded to the diagnostic classification of the participants being assessed. Patients also received comprehensive neuropsychological assessments, including the following standardized tests: Rey Auditory Verbal Learning Test and Free and Cued Selective Reminding Test (verbal memory and learning); Rey Complex Figure (constructional ability and visual memory); Trail Making Test A and B (conceptual tracking and shifting abilities); Stroop test (working memory and attention); Colored Progressive Matrices (abstract thinking); FAS test and Animal Naming (phonemic and semantic verbal fluency). All participants were tested in Italian without the need for an interpreter. Laboratory tests and structural neuroimaging were also performed based on clinical indications.

A team of experienced clinicians assessed the participants’ cognitive and functional status using evidence gathered from all clinical and investigative results, excluding the BASIC and BASIC-Q assessments. They classified the participants according to established criteria into the following diagnostic categories: (1) cognitively intact (or SCD), (2) MCI, and (3) dementia. Participants with normal age, sex, and education-adjusted cognitive performance at the neuropsychology assessment were defined as SCD [25]. MCI was diagnosed based on the criteria established by the International Working Group [26]. For dementia, diagnoses were made according to the 5th edition of the Diagnostic and Statistical Manual of Mental Disorders (i.e., major neurocognitive disorder) [27], with clinical research criteria applied for specific subtypes of dementia disorders [28–32].

### Statistical analysis

The characteristics of the study population were summarized using medians with first and third quartiles (Q1; Q3) or absolute numbers with proportions, as appropriate. Differences between the three diagnostic groups were determined using the Kruskal-Wallis test for continuous variables and the χ^2^ test for categorical variables. Effect sizes were calculated as η^2^ and interpreted according to common criteria [33].

Spearman’s coefficients were used to measure the direction and strength of the correlation of the BASIC and BASIC-Q with participants’ sociodemographic and clinical characteristics (i.e., age, education, and MMSE). Education was operationally defined as the number of completed years of schooling. Fully adjusted linear regression models were conducted to explore the association of BASIC and BASIC-Q (dependent variables of interest) with age, sex, education, and cognitive impairment.

A logistic regression analysis explored the association between BASIC and BASIC-Q (independent variables of interest) and cognitive impairment (MCI or dementia) as the dependent variable.

To evaluate diagnostic accuracy, we used a receiver operating characteristic (ROC) analysis to estimate the areas under the curve (AUC), sensitivity, specificity, and positive and negative likelihood ratios (LR + and LR-) of the BASIC and BASIC-Q. The expert clinical consensus diagnosis of cognitive impairment, which included MCI or dementia, served as the reference standard. The cutoff scores for cognitive impairment were those originally published for the BASIC (< 20/25) and BASIC-Q (< 17/20) [15, 16], and cutoffs determined by Youden’s index. Secondary ROC analyses were conducted to test the accuracy of the two instruments in discriminating between dementia vs. no cognitive impairment and MCI vs. no cognitive impairment. AUC values were compared using a nonparametric approach for correlated ROC curves [34].

The analyses were performed with SPSS version 28.0 for Mac and R (version 4.4.1). A p-value < 0.05 (two-tailed) was considered statistically significant.

## Results

### Characteristics of participants

A total of 246 participants were recruited from the two CCDDs (173 in Rome and 73 in Milan). They had a mean (SD) age of 74.6 (10.1) years, a median (Q1; Q3) educational level of 10.0 (5.0; 13.0) years, and 55.7% were women. All participants were native-born Italians, except for five individuals who had a migration background: three from Egypt, one from Ireland, and one from the Philippines. The median (Q1; Q3) MMSE, BASIC, and BASIC-Q scores were 23.0 (18.0; 27.0), 13.0 (8.0; 17.8), and 12.0 (7.0; 15.0), respectively.

The study population included 42 (17.1%) cognitively intact individuals, 88 (35.8%) with MCI, and 116 (47.1%) with dementia. Among those with dementia, 61 (52.6%) were diagnosed with Alzheimer’s disease (AD), 29 (25.0%) with mixed dementia, 13 (11.2%) with vascular dementia, six (5.2%) with Lewy body dementia, two (1.7%) with frontotemporal dementia, and five (4.3%) with dementia of other etiologies. The sociodemographic and clinical characteristics of participants are shown in Table 2. No differences were found between the samples referred to the two CCDDs.

**Table 2:** Sociodemographic and clinical characteristics of participants according to their cognitive status

	Cognitively intact (*n* = 42)	MCI (*n* = 88)	Dementia (*n* = 116)	*p*
Age, years				< 0.001
Median (Q1; Q3)	66.5 (57.3; 77.0)	75.0 (69.0; 80.0)	79.0 (75.0; 83.0)	
Range	44.0–91.0	28.0–89.0	57.0–93.0	
Sex, N (%)				
Women	24 (57.1)	45 (51.1)	68 (58.6)	0.56
Men	18 (42.9)	43 (48.9)	48 (41.4)	
Education, years				< 0.001
Median (Q1; Q3)	13.0 (8.0; 13.0)	13.0 (8.0; 15.3)	8.0 (5.0; 13.0)	
Range	4.0–18.0	3.0–18.0	0–18.0	
Migration background, N (%)	0 (0.0)	3 (3.4)	2 (1.7)	0.63
MMSE				< 0.001
Median (Q1; Q3)	29.0 (28.0; 30.0)	25.5 (22.0; 27.0)	18.0 (14.0; 20.0)	
Range	25.0–30.0	1.0–30.0	2.0–28.0	
BASIC (*n* = 246)				< 0.001
Median (Q1; Q3)	23.0 (20.0; 24.0)	14.0 (11.0; 17.0)	9.0 (6.0; 13.0)	
Range	10.0–25.0	4.0–23.0	0–21.0	
BASIC-Q (*n* = 173)				< 0.001
Median (Q1; Q3)	20.0 (17.8; 20.0)	13.0 (11.0; 15.0)	7.5 (6.0; 10.8)	
Range	9.0–20.0	3.0–20.0	0–19.0	

**Fig. 1:**
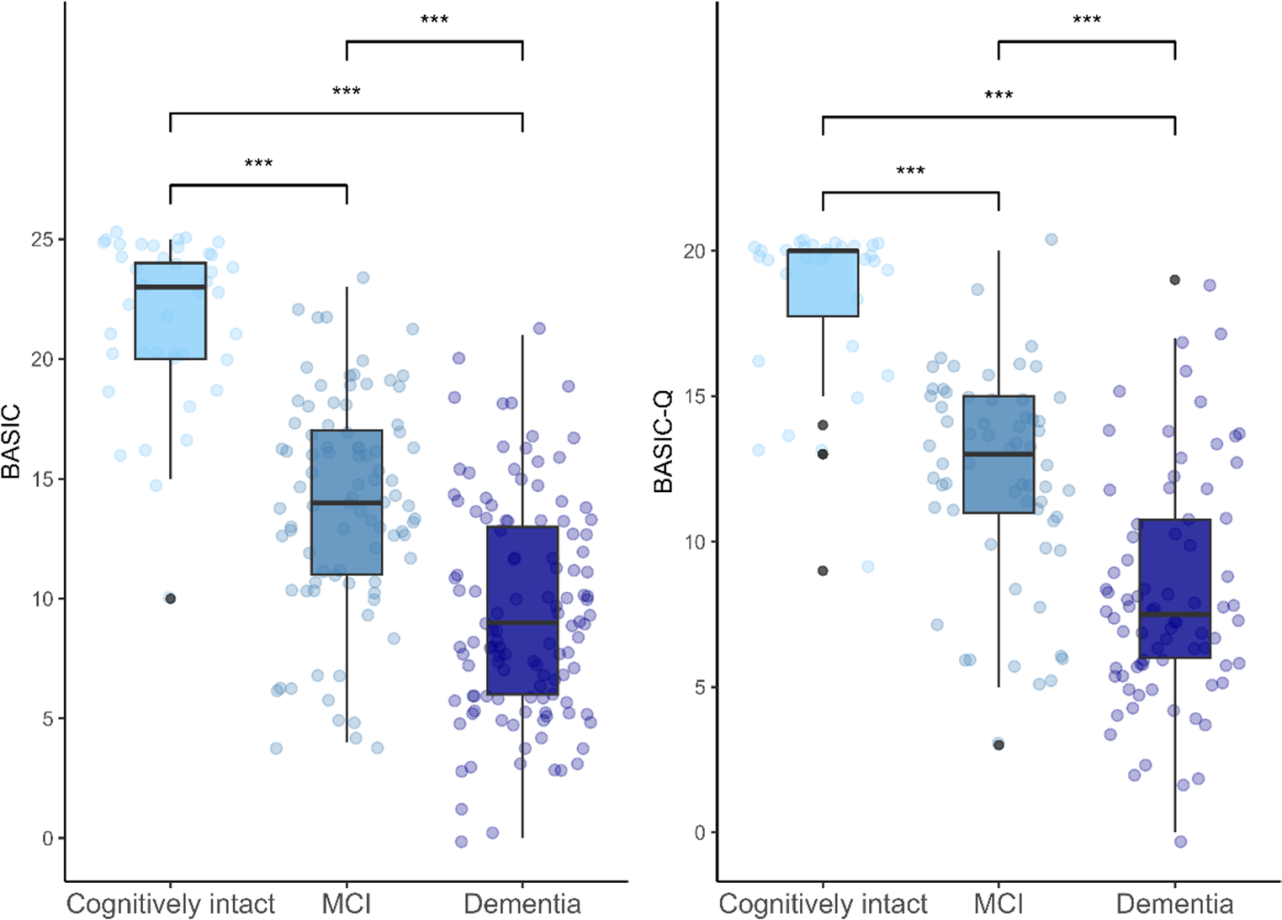
Boxplots of BASIC and BASIC-Q score distributions according to participants’ cognitive status. Group differences based on the Kruskal-Wallis test with post-hoc comparisons. ***p < 0.001

Statistically significant differences were found for BASIC and BASIC-Q scores between the three diagnostic groups, being lower (*p* < 0.001) in participants with dementia and MCI than in cognitively intact participants (Table 2; Fig. 1). Effect sizes for comparisons across the three groups were large for both BASIC (η^2^ = 0.51) and BASIC-Q (η^2^ = 0.52).

### Influence of sociodemographic characteristics

**Fig. 2:**
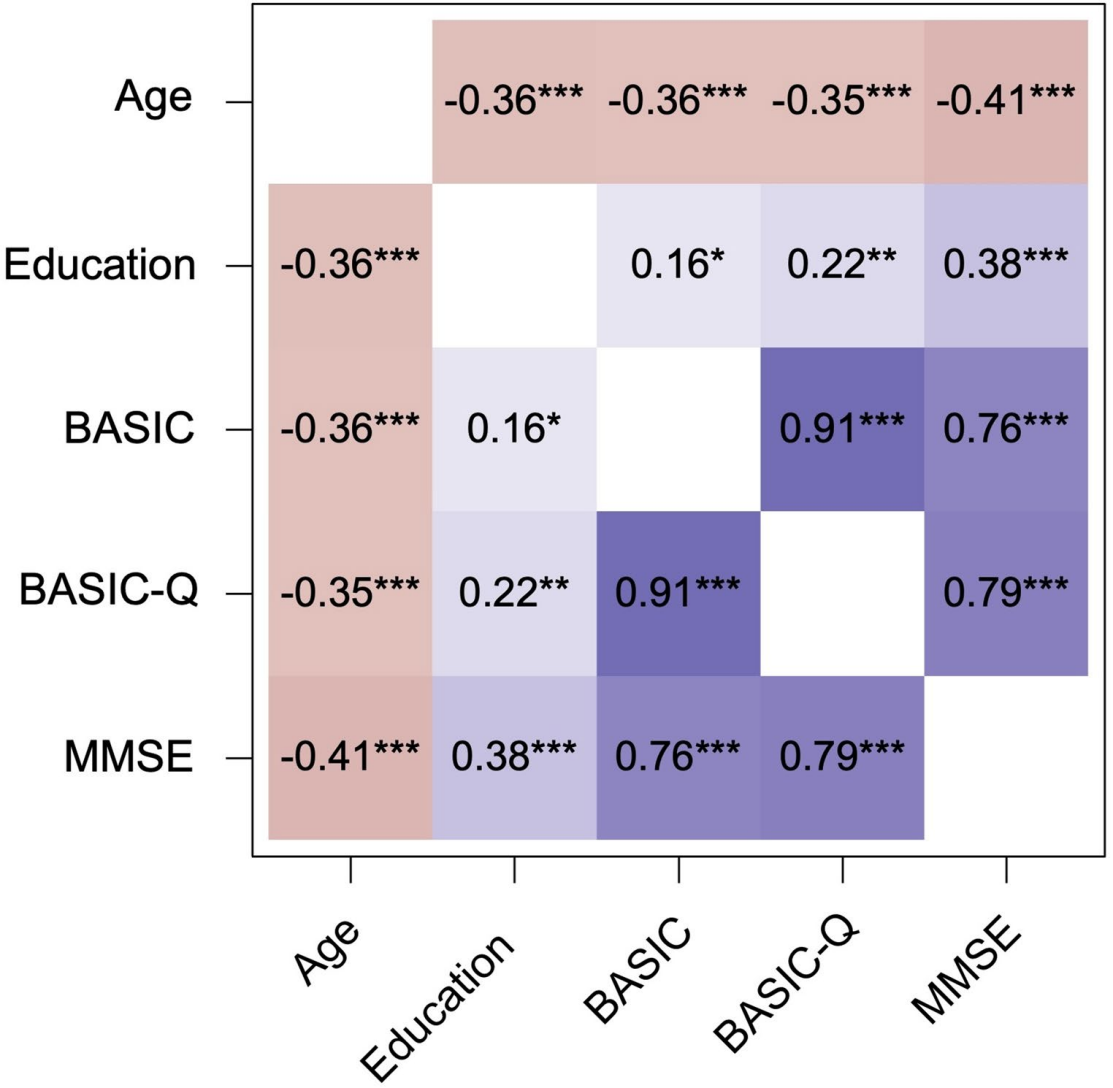
Matrix of Spearman’s correlations of BASIC and BASIC-Q with other sociodemographic and cognitive variables. *p < 0.05; ** p < 0.01; ***p < 0.001

BASIC and BASIC-Q scores exhibited weak but significant, negative correlations with age (BASIC: Spearman’s rho = −0.36; BASIC-Q: rho = −0.35) and education (BASIC: rho = 0.16; BASIC-Q: rho = 0.22) (Fig. 2). In adjusted linear regression models, age remained significantly and negatively associated with BASIC (B = −0.12, 95% CI = −0.19, −0.05; *p* < 0.001) and BASIC-Q (B = −0.08, 95% CI = −0.15, −0.01; *p* = 0.03) scores. No association was found between education and the two test scores (all p values > 0.05), whereas male sex was associated with higher BASIC-Q scores (B = 1.44, 95% CI = 0.25, 2.63; *p* = 0.02) (Table 3).

**Table 3:** Linear regression models exploring the association of BASIC and BASIC-Q (dependent variables of interest) with traditional cognitive confounders

	BASIC			BASIC-Q		
	B	95% CI	*p*	B	95% CI	*p*
Age	−0.12	−0.19, −0.05	< 0.001	−0.08	−0.15, −0.01	0.03
Sex (M)	0.90	−0.29, 2.09	0.14	1.44	0.25, 2.63	0.02
Education	0.03	−0.11, 0,17	0.69	0.09	−0.04, 0.23	0.17
Cognitive impairment*	−9.21	−10.87, −7.55	< 0.001	−7.65	−9.25, −6.04	< 0.001

*Cognitive impairment: MCI or dementia

### Diagnostic accuracy

In logistic regression models adjusted by age, sex, and education, BASIC and BASIC-Q scores were negatively associated with cognitive impairment (MCI and dementia) (BASIC: OR = 0.58, 95% CI = 0.49, 0.69; *p* < 0.001. BASIC-Q: OR = 0.53, 95% CI 0.42, 0.67; *p* < 0.001) (Online Resource 3).

**Fig. 3:**
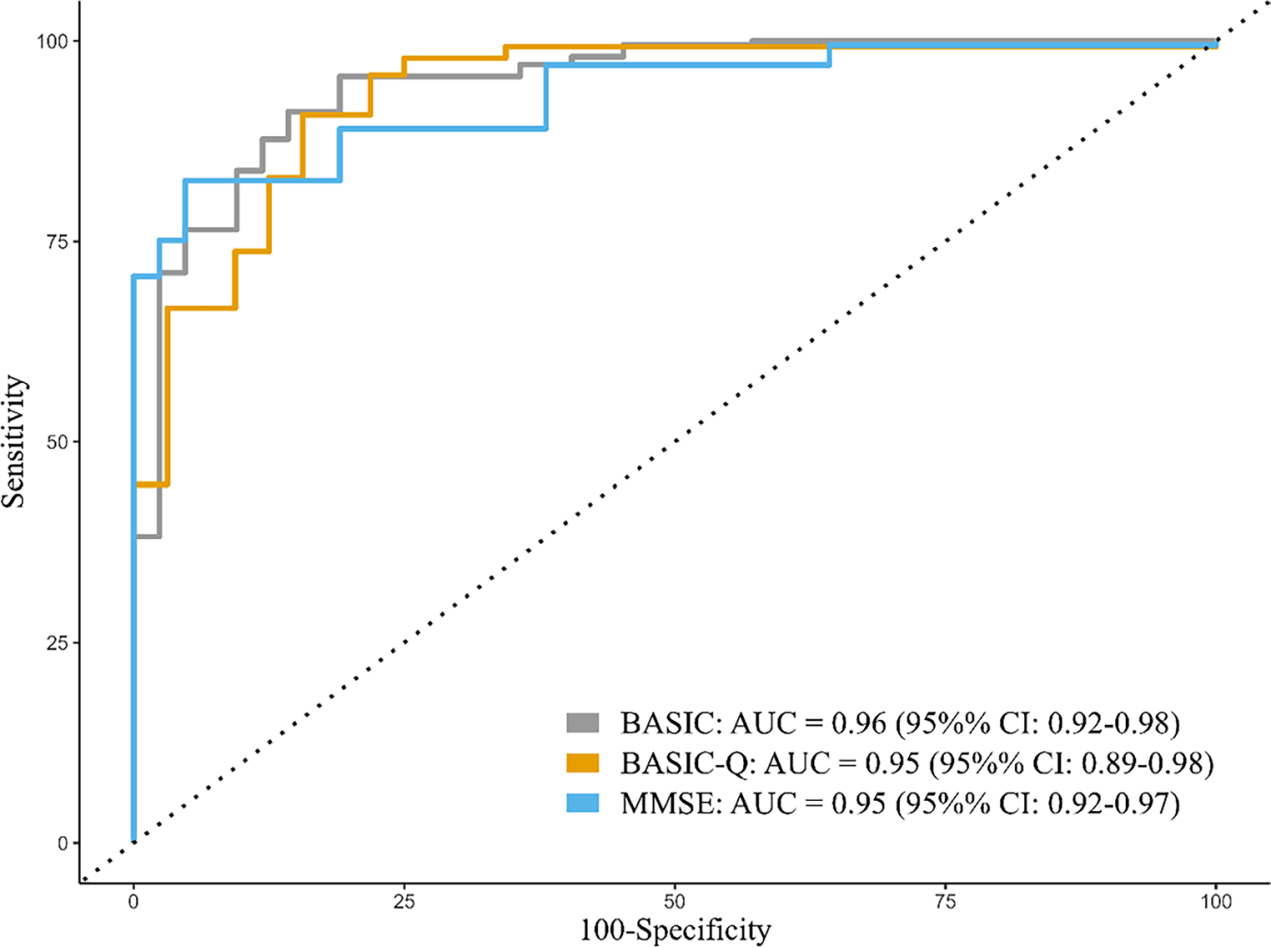
Receiver operating characteristics (ROC) curves for BASIC, BASIC-Q, and MMSE for detecting cognitive impairment (MCI or dementia)

ROC curve analysis indicated that both BASIC and BASIC-Q had high accuracy for discriminating patients with cognitive impairment from cognitively intact participants (BASIC: AUC = 0.96, 95% CI = 0.92, 0.98; BASIC-Q: AUC = 0.95, 95% CI = 0.89, 0.98). Based on pairwise comparisons of the AUCs using a paired-sample design, the two instruments’ discriminative validity was comparable (*p* > 0.05) with that of the MMSE (AUC = 0.95, 95% CI = 0.92, 0.97) (Fig. 3).

A cutoff score of < 19/25 on BASIC provided optimal discrimination between cognitively impaired and cognitively intact participants, with excellent specificity (0.91), sensitivity (0.86), LR+ (6.38), and LR- (0.10) (Table 4). Accordingly, the BASIC-Q had an optimal specificity (0.91), sensitivity (0.84), LR+ (5.81), and LR- (0.11) at a cutoff score of < 16/20 (Table 4).

**Table 4:** Discriminative accuracy of the BASIC and BASIC-Q

Cognitive impairment vs. no cognitive impairment						
Test	AUC (95% CI)	Cutoff	Sensitivity (95% CI)	Specificity (95% CI)	LR+ (95% CI)	LR- (95% CI)
BASIC	0.96 (0.92, 0.98)	< 19/25*	0.91 (0.86, 0.95)	0.86 (0.71, 0.95)	6.38 (3.04, 13.40)	0.10 (0.07, 0.16)
		< 20/25^§^	0.96 (0.93, 0.98)	0.81 (0.69, 0.93)	5.02 (3.60, 7.00)	0.05 (0.04, 0.08)
BASIC-Q	0.95 (0.89, 0.98)	< 16/20*	0.91 (0.85, 0.95)	0.84 (0.67, 0.95)	5.81 (2.59, 13.02)	0.11 (0.06, 0.19)
		< 17/20^§^	0.96 (0.92, 0.99)	0.78 (0.64, 0.92)	4.38 (2.98, 6.43)	0.05 (0.04, 0.08)
**Dementia vs. no cognitive impairment**						
Test	AUC (95% CI)	Cutoff	Sensitivity (95% CI)	Specificity (95% CI)	LR+ (95% CI)	LR- (95% CI)
BASIC	0.98 (0.95, 1.00)	< 16/25*	0.90 (0.83, 0.95)	0.95 (0.84, 0.99)	18.83 (4.86, 72.91)	0.11 (0.06, 0.19)
BASIC-Q	0.97 (0.94, 0.99)	< 13/20*	0.83 (0.73, 0.91)	0.97 (0.84–1.00.84.00)	26.67 (3.86, 184.03)	0.17 (0.10, 0.28)
**MCI vs. no cognitive impairment**						
Test	AUC (95% CI)	Cutoff	Sensitivity (95% CI)	Specificity (95% CI)	LR+ (95% CI)	LR- (95% CI)
BASIC	0.92 (0.86, 0.97)	< 20/25*	0.92 (0.84, 0.97)	0.81 (0.66, 0.91)	4.83 (2.58, 9.04)	0.10 (0.05, 0.20)
BASIC-Q	0.91 (0.84, 0.97)	< 17/20*	0.95 (0.87, 0.99)	0.78 (0.60, 0.91)	4.35 (2.26, 8.40)	0.06 (0.02, 0.19)

* Optimal cutoff score in the present sample based on Youden’s J

^§^ Originally published cutoff score

Both tests accurately discriminated between dementia or MCI (considered separately) vs. no cognitive impairment (Table 4).

To assess the impact of socio-demographic adjustments on classification accuracy, we calculated age-adjusted scores for the BASIC across the entire sample. However, this adjustment did not significantly influence overall classification accuracy, as indicated by p-values ranging from 0.4 to 0.7 in pairwise DeLong comparisons (Online Resource 4).

The accuracy of BASIC and BASIC-Q remained unchanged in sensitivity analyses, excluding the five participants with a migration background.

## Discussion

The current study confirms that the Italian versions of BASIC and BASIC-Q accurately distinguish between patients with and without cognitive impairment. Using these brief and easy-to-administer cognitive instruments may aid in the timely identification of cognitive disorders within the Italian population.

In our sample, optimal cutoff scores of < 19/25 for the BASIC and < 16/20 for the BASIC-Q discriminated patients with MCI or dementia from cognitively intact individuals with SCD with excellent accuracies, sensitivities, specificities, and likelihood ratios. These findings align with those already documented in both monocultural and multicultural patient populations and different care settings [15–17, 19–21]. Interestingly, the discriminative validity of BASIC and BASIC-Q was comparable to that of the MMSE, the most widely adopted cognitive screening tool. Nevertheless, the MMSE requires a longer time for administration and is more significantly affected by educational and cultural biases [35]. Moreover, in the two CCDDs, the MMSE was part of the diagnostic workup, and clinicians making the diagnoses were not blind to the MMSE results. This likely introduces the risk of confirmation bias and related artificial inflation of the diagnostic accuracy of the MMSE.

Our study also confirms previous evidence that BASIC and BASIC-Q are only slightly influenced by age but not by education. This characteristic may be beneficial for CALD individuals but also for people born and raised in Italy, where in 2011, 2.3% of the population over 55 years was illiterate, 7.8% lacked any educational qualification, and 41.3% had only completed primary school education [36]. BASIC-Q scores were found to be positively associated with male sex. However, this association is likely influenced by the fact that male participants tended to be younger and more educated than their female counterparts.

 The BASIC and BASIC-Q instruments should be included in the toolkit available to specialized centers as effective options for the initial evaluation of individuals with cognitive disorders. They can also be considered for the assessment of CALD individuals, such as migrants or those with lower education levels. In this regard, the national survey of CCDDs, conducted as part of the Immidem project, revealed that only a marginal percentage of centers (1.2%) offer cross-cultural tests [37]. Furthermore, cognitive assessments are rarely adjusted to account for the cultural background of the individuals being evaluated, with a high risk of dementia misclassification.

These two tests could be even more effectively utilized at the community and primary care levels to promptly identify individuals experiencing cognitive decline. This timely identification is crucial for recognizing reversible causes, promoting lifestyle changes, improving referrals to specialized services, and ensuring access to both pharmacological and non-pharmacological treatments (such as cognitive stimulation), counseling, and community support services [38]. The Italian guideline for the Diagnosis and Treatment of Dementia and Mild Cognitive Impairment does not support case-finding strategies and recommends the use of several brief cognitive tests for initial assessment in non-specialist settings [39]. Among them, the GPCOG [11] is currently the most adopted test by Italian primary care physicians. However, most of the included items, such as recalling a name and address, orientation in time, clock-drawing, and talking about a recent event, can be affected by low education or limited linguistic abilities [40, 41].

The main limitation of the present study is that the patient population, entirely composed of patients referred to university CCDDs, may not be representative of a community or primary care setting. Specifically, it included only a few individuals with low educational levels and a migration background. Although the accuracy of BASIC and BASIC-Q has already been proven in these settings [16, 18], future studies are needed to confirm their accuracy in Italian primary care as well as in multicultural populations. We did not include patients with affective/anxiety disorders, who are commonly encountered in both memory clinics and primary care settings, and are at high risk of clinical misclassification. In this regard, the BASIC and BASIC-Q, particularly the ratio between objective performance and subjective complaints, have already been shown to discriminate well between patients with cognitive impairment and affective disorders [21]. Another possible shortcoming is that the agreed-upon criteria for the three diagnostic groups may have been applied differently by the two participating sites. The limited sample size did not allow us to ascertain the accuracy of BASIC and BASIC-Q in discriminating specific etiological diagnoses and phenotypic presentations of neurocognitive disorders. Indeed, it can be hypothesized that, based on their structure, the two tools may be particularly sensitive in detecting problems with memory and orientation, thus supporting the preferential identification of patients with AD and amnesic presentations. However, the study population included a large proportion of non-AD patients. Moreover, a previous study had already shown that the BASIC has high discriminative validity for both AD and non-AD dementia [20]. Finally, we failed to provide relevant psychometrics (e.g., test-retest reliability and convergent validity) for the two tests [42]. Nevertheless, some of these properties, such as test-retest reliability, had already been established in previous studies [18, 20].

This study also has several strengths. The two instruments were translated and adapted by following a rigorous process. The raters who administered the BASIC and BASIC-Q received specific training and were unaware of the diagnostic status of participants. The classification of patients by cognitive status was based on a comprehensive diagnostic workup and established diagnostic criteria.

In conclusion, the BASIC and BASIC-Q are concise and effective tools for identifying individuals with cognitive impairment for whom a further diagnostic assessment should be considered. However, their accuracy must be confirmed in primary care settings and in multicultural populations. To promote their dissemination and adoption on a large scale, the Italian versions of the two instruments will be made freely available on the Immidem website (https://immidem.it/) and represent the object of targeted training activities for healthcare professionals within the Immidem project.

## Supplementary Information

Below is the link to the electronic supplementary material.

Supplementary Materials 1 (DOCX 535 KB)
