# Supplementary material for: Validation of the brief assessment of impaired cognition (BASIC) and brief assessment of impaired cognition questionnaire (BASIC-Q) in Italy
Source: Neurol Sci. 2026 Mar 9;47(4):332. doi: 10.1007/s10072-025-08691-w (PMC12968103; doi:10.1007/s10072-025-08691-w)
Supplement: Supplementary file 1 [file 10072_2025_8691_MOESM1_ESM.docx]

Online Resources

Online Resource 1. The Italian version of the Brief Assessment of Impaired Cognition (BASIC)

Immagine che contiene testo, ricevuta, schermata, Carattere

Il contenuto generato dall'IA potrebbe non essere corretto. 

Online Resource 2. The Italian version of the Brief Assessment of Impaired Cognition Questionnaire (BASIC-Q)

Immagine che contiene testo, ricevuta, schermata, numero

Il contenuto generato dall'IA potrebbe non essere corretto.

Online Resource 3. Logistic regression models exploring the association of BASIC and BASIC-Q (independent variables of interest) with cognitive impairment (MCI or dementia, bivariate dependent variable of interest).

|  | Cognitive impairment |
| --- | --- |
|  | OR | 95% CI | p |
| Age | 1.02 | 0.97, 1.07 | 0.51 |
| Sex (M) | 1.23 | 0.43, 3.51 | 0.70 |
| Education | 1.04 | 0.92, 1.19 | 0.53 |
| BASIC | 0.58 | 0.49, 0.69 | <0.001 |
|  |
|  | OR | 95% CI | p |
| Age | 1.02 | 0.96, 1.09 | 0.58 |
| Sex (M) | 1.64 | 0.49, 5.50 | 0.42 |
| Education | 1.04 | 0.89, 1.22 | 0.61 |
| BASIC-Q | 0.53 | 0.42, 0.67 | <0.001 |

Online Resource 4. Comparison of the discriminative accuracy of the unadjusted and age-adjusted BASIC.

|  | Unadjusted BASIC | Age-adjusted BASIC |  |
| --- | --- | --- | --- |
|  | AUC (95% CI) | AUC (95% CI) | p* |
| Cognitive impairment vs. no cognitive impairment | 0.96 (0.92, 0.98) | 0.95 (0.91, 0.98) | 0.6 |
| Dementia vs. no cognitive impairment | 0.98 (0.95, 1.00) | 0.98 (0.95, 1.00) | 0.7 |
| MCI vs. no cognitive impairment | 0.92 (0.86, 0.97) | 0.91 (0.85, 0.96) | 0.4 |

*p-values from pairwise DeLong comparisons
